# Unveiling the crucial role of iron mineral phase transformation in antimony(V) elimination from natural water

**DOI:** 10.1016/j.eehl.2023.07.006

**Published:** 2023-07-24

**Authors:** Xiaoyun Liu, Yunyan Wang, Hongrui Xiang, Jiahui Wu, Xu Yan, Wenchao Zhang, Zhang Lin, Liyuan Chai

**Affiliations:** a School of Metallurgy and Environment, Central South University, Changsha 410083, China; b State Key Laboratory of Advanced Metallurgy for Non-ferrous Metals, Changsha 410083, China; c Chinese National Engineering Research Center for Control & Treatment of Heavy Metal Pollution, Changsha 410083, China

**Keywords:** Ferrihydrite transformation, Water environment, Sb(V) level, Removal mechanism, Pollution control

## Abstract

Antimony (Sb) in natural water has long-term effects on both the ecological environment and human health. Iron mineral phase transformation (IMPT) is a prominent process for removing Sb(V) from natural water. However, the importance of IMPT in eliminating Sb remains uncertain. This study examined the various Sb–Fe binding mechanisms found in different IMPT pathways in natural water, shedding light on the underlying mechanisms. The study revealed that the presence of goethite (Goe), hematite (Hem), and magnetite (Mag) significantly affected the concentration of Sb(V) in natural water. Elevated pH levels facilitated higher Fe content in iron solids but impeded the process of removing Sb(V). To further our understanding, polluted natural water samples were collected from various locations surrounding Sb smelter sites. Results confirmed that converting ferrihydrite (Fhy) to Goe significantly reduced Sb levels (<5 μg/L) in natural water. The emergence of secondary iron phases resulted in greater electrostatic attraction and stabilized surface complexes, which was the most likely cause of the decline of Sb concentration in natural water. The comprehensive findings offer new insights into the factors governing IMPT as well as the Sb(V) behavior control.

## Highlights

• An attractive and efficient approach to achieve Sb(V) pollution control in natural water by IMPT was proposed.
• The residual Sb could be barely detected at pH 5.5 and 7 (only 1.747 μg/L) after IMPT process.
• Intensified electrostatic attraction and inner-sphere bidentate binuclear complexation contributed to the significant decrease of Sb(V).

## Graphical abstract

**Figure:**
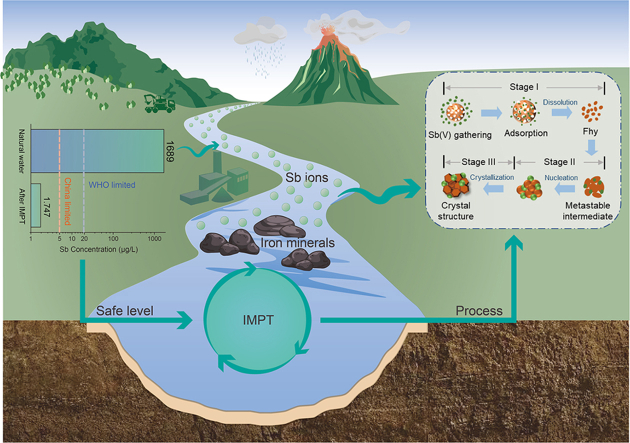

## 1 Introduction

Antimony (Sb) pollution has garnered significant global attention due to its undesired properties, such as uncontrolled mobility, high toxicity, potential carcinogenicity, and negative impact on the ecological environment and human health [[1], [2], [3]]. As early as the 1970s, Sb has been listed as a pollutant of priority interest by the European Union and the United States Environmental Protection Agency. The World Health Organization and China have established criteria for the maximum acceptable levels of Sb in drinking water, set at 20 μg/L and 5 μg/L, respectively [[4], [5], [6]]. Natural causes of Sb release include leaching of wet deposition and volcanic eruptions [7], while artificial exploitation has become a dominating anthropogenic pathway for discharging a large amount of Sb into the water environment [[8], [9], [10]]. The environmental problems are getting exacerbated by the powerful influence of human activities and the widespread application of Sb products [11,12]. China, the country with the most abundant Sb resources, accounted for a significant proportion of global anthropogenic Sb emissions [13]. It has been reported that the Sb level in the water near the Xikuangshan mine, the world’s largest Sb deposit, can reach 224–6,384 μg/L [[14], [15], [16]].

Iron minerals phase transformation (IMPT), especially the transformation of metastable Fe(III) oxyhydroxides, is considered a prominent process of Sb removal in wastewater [17]. In the past decades, researchers have focused on exploring the mobility and release of potentially toxic elements (PTEs) in the transformation of Fe-PTEs co-precipitate [18,19]. However, the multiple removal mechanisms of Sb in IMPT remain unclear, and the derived health problems are easily overlooked. Sb is a sulfhydryl toxin that binds to certain enzymes in humans, disrupting tissue metabolism, damaging the brain and genital system, and causing illnesses and diseases such as pneumoconiosis, chronic bronchitis, and dermatitis [[20], [21], [22]]. To curb the increasing risk of Sb contamination in natural water, it is imperative to investigate the control of Sb during the IMPT process, which can help mitigate the associated human health concerns.

The geochemical and environmental behavior of Sb is closely related to the intricate IMPT. Most iron-bearing minerals exhibit high reactivity and can strongly adsorb Sb(V) to form surface complexes [23]. Ferrihydrite (Fhy), a typical precursor to most iron oxyhydroxides, may coalesce into aggregates or transform into a variety of more thermodynamically stable iron phases such as goethite (Goe) and hematite (Hem) [24]. IMPT is greatly influenced by pH, and the binding ability towards PTEs decreases significantly when the pH surpasses the point of zero charge (pH_pzc_) [25]. Extensive studies have reported that iron minerals might lead to the variation of PTEs toxicity and speciation in the water environment through surface adsorption [26,27]. Nevertheless, the multiple surface-binding mechanisms of Sb(V) and iron oxyhydroxides in complicated mineralization pathways remain elusive, which poses challenges in gaining deeper insights into the influence of the IMPT process on PTEs behavior control.

Herein, this study involves three objectives: 1) To investigate the impact of the IMPT process on the behavior of PTEs pollution control. This was achieved by subjecting Sb(V) to the IMPT process under multiple pH values, and conducting some characterization like X-ray photoelectron spectroscopy (XPS) and X-Ray absorption fine structure (EXAFS); 2) To unveil the mechanisms of Sb–Fe binding in IMPT process and the removal behavior of Sb(V), thus providing a promising method to control Sb pollution in water environment; 3) To collect representative environmental water samples from Sb smelter sites with serious water pollution, to verify the critical role of IMPT in alleviating PTEs pollution in natural water and provide some new insights.

## 2 Materials and methods

### 2.1 Chemicals and materials

Fe(NO_3_)_3_·9H_2_O, FeSO_4_·7H_2_O, KOH, HCl, and HNO_3_ were purchased from Sinopharm Chemical Reagent Co., Ltd. KSbO_6_H_6_, 2-(N-morpholino)ethanesulfonic acid (MES), 3-(N-morpholino)propanesulfonic acid (MOPS), 4-(2-hydroxyethyl)piperazine-1-ethanesulfonic acid hemisodium salt (HEPES), 2-(cyclohexylamino)ethanesulfonic acid (CHES), and 3-(cyclohexylamino)-1-propanesulfonic acid (CAPS) were obtained from Shanghai Macklin Biochemical Co., Ltd. All chemicals used in this study were of analytical grade and were used without further purification. The ultrapure water used in all experiments of this work was purified through a Millipore system (≥18 MΩ).

### 2.2 Synthesis of Fhy and transformation experiments

Fhy was synthesized according to the method described by Schwertzmann and Cornell [28]. Fhy precipitation was induced by dissolving 40 g Fe(NO_3_)_3_·9H_2_O in 500 mL deionized water and adjusting the pH to 7–8 with 8 M KOH. The resulting suspension was centrifuged, and then washed with deionized water until free from electrolytes, freeze-dried, sieved through 200 mesh, and stored as a solid product. This method yielded approximately 10 g of 2-line Fhy, a poorly crystalline or amorphous phase of iron (III) oxide characterized by its disordered atomic arrangement and two typical diffraction peaks in the XRD pattern.

To investigate the effect of the IMPT process on Sb in a variety of environmental water samples, phase transformation experiments of Fhy were conducted at pH 4, 5.5, 7, 8, 10, and 11 over a reaction period of 24 h. The mixed solution containing 0.2 g of Fhy, 10,000 μg/L Sb(V), 3 mM FeSO_4_·7H_2_O, and 0.1 mol/L buffer solutions was added into the flasks. Then, the flasks were sealed and shaken at 60 °C with a speed of 150 rpm. The FeSO_4_·7H_2_O solution was prepared in diluted and N_2_-purged H_2_SO_4_. The buffer solutions at pH 5.5, 7, 8, 10, and 11 were prepared with MES, MOPS, HEPES, CHES, and CAPS buffer solutions to maintain pH stability. The solution pH was adjusted using 1% H_2_SO_4_ and 0.5–8 mol/L NaOH. To investigate the Fe content of transformed products, 37% HCl was used as a reagent to completely dissolve these samples.

To investigate the influence of the reaction time, the suspensions were respectively collected from the flasks after 0, 10, 30, 60, 240, 480, and 1,440 min. The initial aqueous Sb(V) concentration in the suspensions was 10,000 μg/L. The whole experiment was pumped with N_2_ to avoid Fe(II) oxidation. The samples were then sealed and transferred under vacuum conditions. The solid phase samples obtained after the reaction were collected, dried in a freeze dryer for 24 h, ground, and sealed storage at room temperature for further characterization and chemical analyses. Subsequently, the solutions were separated by 0.22 μm membrane filtration, and the Sb(V) concentrations in the filtrates were monitored using inductively coupled plasma-mass spectrometry (ICP-MS, NEXION 2000).

### 2.3 Extraction experiments

0.2 mol/L Na_3_PO_4_ or 37% HCl was used for the extraction experiments to determine the content of Sb(V) in the transformation products [29,30]. The solid/solution ratio was 1:2000 (0.025 g transformation products to 50 mL extractant). The suspensions were shaken for 8 h and then filtered through 0.22 μm membrane for the analysis of the concentrations of Sb(V) in the filtrates using an inductively coupled plasma-optical emission spectrometer (ICP-OES, TCP-5100-VDV).

### 2.4 Fhy transformation experiments in natural water

To explore how IMPT in natural water affects the level of Sb, we examined three representative water samples (1#, 2#, and 3#) around Sb smelter sites and designed the verification experiments of Fhy transformation. The dosage of Fhy was 1 g/L, and the suspensions were respectively collected from the centrifuge tube after 5, 10, 20, 30, 60, 120, 240, 480, and 1,440 min at 25 °C. Other conditions were consistent with the above transformation experiments.

### 2.5 Solid phase analysis

The reacted solid phases were analyzed using X-ray diffraction (XRD), Fourier transform infrared (FTIR), and X-ray photoelectron spectroscopy (XPS). The morphology of the transformation products and element distribution of the solid phases were observed by scanning electron microscope (SEM) and transmission electron microscopy (TEM). The bonding of the products was analyzed using extended X-Ray absorption fine structure (EXAFS). Detailed information is provided in Supplementary Text S1. The configuration of transformation products was optimized by density functional theory (DFT) calculations (Text S2).

## 3 Results and discussion

### 3.1 Phase identification of IMPT process

**Fig. 1:**
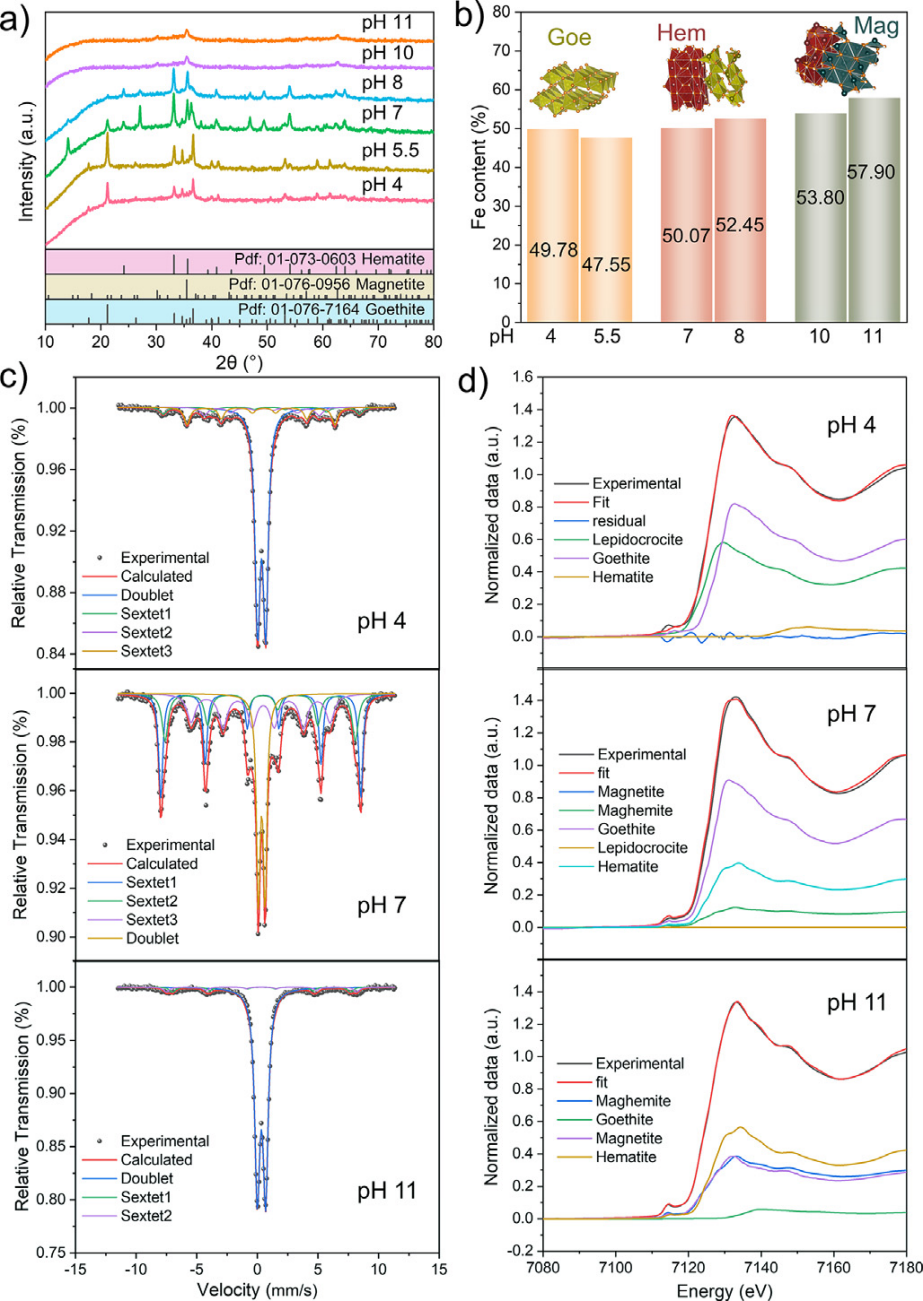
Phase identification of transformation products at different pH conditions. (a) XRD patterns; (b) Fe content of different products; top inset, optimized crystal structure by DFT, showing their crystal form change during precipitation; (c) Mössbauer spectra; (d) LCF analysis. XRD, X-ray diffraction; DFT, density functional theory; LCF, linear combination fitting; Goe, goethite; Hem, hematite; Mag, magnetite.

The conversion of metastable Fhy to stable iron minerals is an indispensable step in the IMPT process. The XRD pattern of 2-line Fhy in Fig. S1 revealed an amorphous structure, with two broad diffraction peaks at ∼34° and ∼61° [31]. Fig. 1a showed the XRD patterns of the transformed products at pH 4, 5.5, 7, 8, 10, and 11 after 24 h of reaction. Goe was the dominant crystalline solid in the precipitates at pH 4 and 5.5. Fhy was predominantly converted to Goe and Hem at pH 7 and 8, while the sample consisted of magnetite (Mag) and Hem at pH 10 and 11. The Fe content of the transformed products was measured by complete dissolution (Fig. 1b). The Fe content of the samples at pH 4 and 11 was 49.78% and 57.90%, respectively. High pH favored the formation of iron solid phase with higher Fe content, and the crystal structure changed from layered to compact [32].

Mössbauer spectroscopy is a powerful technique to further determine the iron solid phase based on its strong sensitivity to iron [33]. The hyperfine splitting field is a crucial parameter exploited to match phase composition (Table S1). The spectrum of the sample at pH 4 consisted of one doublet with a larger area and three sextets with a small area. According to the previous research, γ-FeOOH is a paramagnetic doublet, while *α*-FeOOH is a hyperfine sextet, and both can be distinguished by the presence or absence of hyperfine fields [34,35]. *α*-Fe_2_O_3_ and γ-Fe_2_O_3_ are difficult to distinguish using the Mössbauer spectra, where the hyperfine field of Fe_2_O_3_ A-site is stronger than Fe_2_O_3_ B-site [36]. Sextet 2 was identified as Mag because its hyperfine field was slightly stronger than that of Hem, and the doublet was Fe(OH)_2_ [37]. These samples possessed a single sextet with a wide magnetic splitting (∼50 T) and a low center shift (0.37–0.38 mm/s), which were the characteristic of Hem. The sextet was formed due to electron hopping within the octahedral lattice and corresponds to Fe^2+^ and Fe^3+^ (Fig. 1c) [38].

The quantitative analysis of iron solid phases at pH 4, 7, and 11 can be further confirmed by linear combination fitting (LCF) (Fig. 1d). All experimental signals were acquired by fitting of shell layers and compared with the theoretical signal. The first shell layer signal exhibited the strongest intensity due to the strong Fe–O interactions, conforming to the relevant contributions of the first atomic shells [39]. In all graphs, the *k*^3^-weighted extracted EXAFS data appear structured and of high quality, with the theoretical curves matching well with the experimental data. LCF results indicated that approximately 56.6% of Goe at pH 4 was formed, so were 62.8% and 27.7% of Goe and Hem at pH 7, 27.4% and 39.4% of Mag and Hem at pH 11, respectively (Table S2). The overall results were consistent with the XRD and Mössbauer spectroscopy. Based on the above analysis, the main phase and trace phase of transformation products were summarized at different pH values (Table S3). These findings proved that Goe, Hem, and Mag were the dominant phase in this system, as well as the formation of a small quantity of lepidocrocite (Lep) and maghemite (Mah).

### 3.2 Chemical bonding characteristics of Sb and Fe

**Fig. 2:**
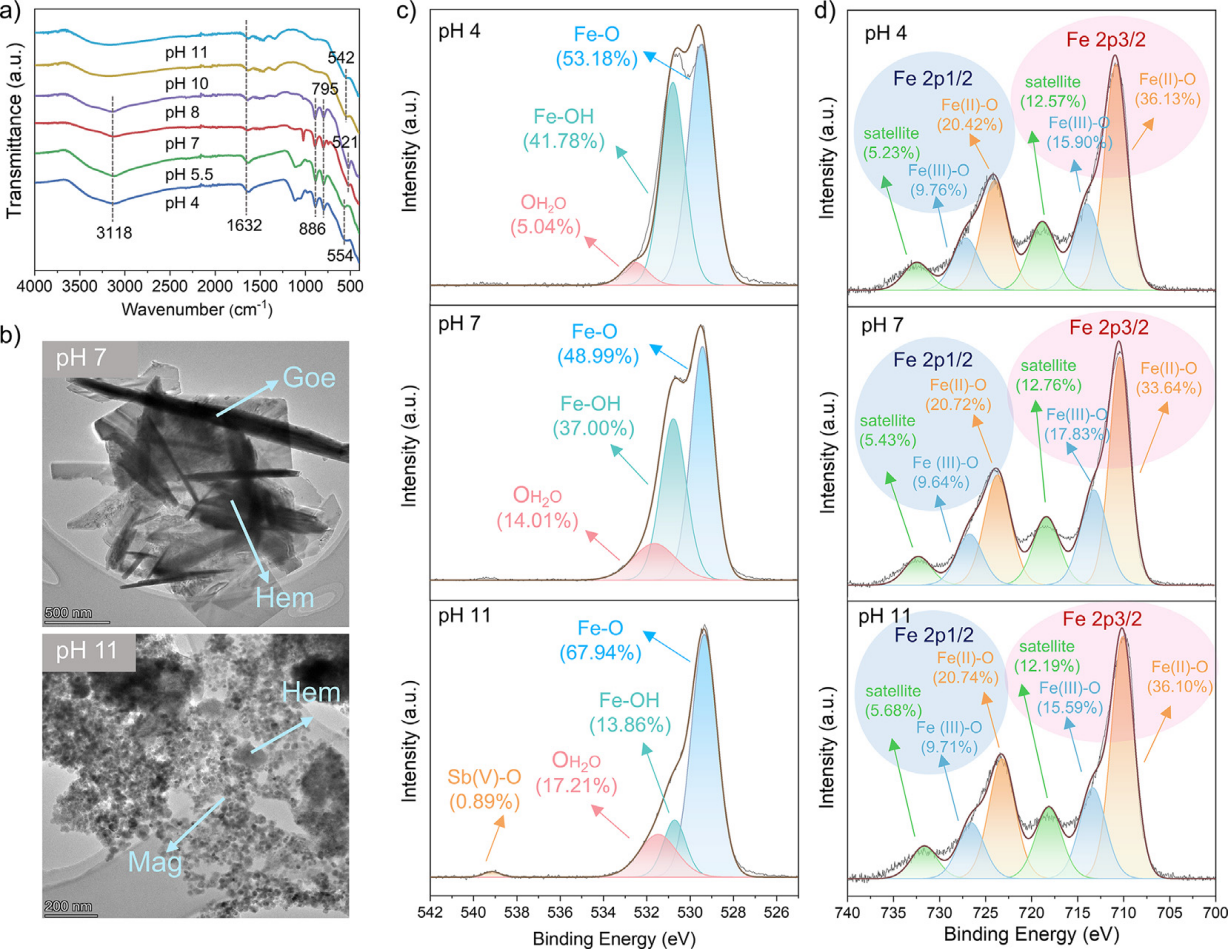
Structure characterization of the transformation products. (a) FT-IR pattern; (b) TEM images at pH 7 and 11; (c, d) high-resolution spectra of Sb 3d + O 1s and Fe 2p at pH 4, 7, and 11.

The FT-IR spectra of the transformed samples were compared in Fig. 2a. The broad peak located at ∼3,118 cm^−1^ was attributed to the stretching vibration of the Fe–OH band and H_2_O_adsorption_ molecule on the surface of iron minerals [40]. The characteristic peak near 1,632 cm^−1^ was ascribed to the bending vibration of the H–O–H band of H_2_O molecules, corresponding to the physisorbed water on the secondary minerals [41]. The signature peaks at 886 cm^−1^ and 795 cm^−1^ were produced by the in-plane and out-of-plane deformation vibration of Fe–OH, indicating the formation of Goe [42]. A remarkable characteristic peak of Goe was observed as the dominant phase at pH 4 and 5.5. Besides Goe, another thermodynamically stable crystalline phase, Hem, was formed at pH 7 and 8. The Fe–O band of transformed products was shifted from 552 cm^−1^ to 521 cm^−1^ due to the formation of iron phases with different structures. Among these, the band at 554 cm^−1^ referred to the Fe(III)–O vibration in octahedral sites, and two peaks at 542 cm^−1^ and 521 cm^−1^ were attributed to Fe–O vibration in octahedral and tetrahedral sites [[43], [44], [45]]. This evidence supported that metastable Fhy was transformed into more stable Goe, Hem, and Mag.

The morphologies of obtained iron solid phases were observed by SEM (Fig. S2). The morphology of Fhy changed from an irregular needle shape to a platy shape, and finally to an irregular round shape [46,47]. The microstructures of the minerals were further characterized by TEM (Fig. 2b, Figs. S3 and S4), with Goe occurring as an acicular or needle-shaped crystal [48]. For the transformation products at pH 7, distinct Goe and Hem phases were observed, confirming SEM observations, but the regional distribution of the Sb(V) was not apparent. The presence of a small amount of Sb(V) on the surface of the transformed iron minerals was evident. From the results of local mapping analysis, the content of Sb(V) on the surface of the transformation products at pH 7 and pH 11 were 0.38% and 1.03%, respectively (Table S4).

XPS survey spectra showed the presence of Fe, Sb, O, and C on the surface of transformation products, with characteristic peaks being visible (Fig. S5). The Sb 3d + O 1s high-resolution spectra (Fig. 2c) were divided into three peaks of ∼532.52 eV, ∼530.81 eV, and ∼529.48 eV (Table S5), which were related to oxygen in adsorbed water (${\text{O}}_{{\text{H}}_{2}\text{O}}$), hydroxyl bonded with Fe (Fe–OH) and iron oxide (Fe–O), respectively [49]. According to the peak area, Fe–O accounted for the largest proportion, followed by Fe–OH and ${\text{O}}_{{\text{H}}_{2}\text{O}}$. The Fe–OH could form a strong coordination bond with Sb(V), thus forming stable complexes, indicating that Fe–OH on the surface of iron oxyhydroxides was crucial for the binding of Sb(V). After reaction for 24 h, the –OH content of products increased, corresponding to the formation of Goe (pH from 4 to 5.5). The content of –OH decreased with the increase of –O^2–^, probably due to the formation of Hem (pH from 7 to 8). In addition, when the pH was 10 and 11, the peak of Sb(V)–O was detected on the surface of the transformation products, and the content was 6.51% and 0.89%, respectively. It was speculated that the formation of the Fe–O–Sb coordination was located in the mineral’s surface [50]. The low Sb(V) content used in the experiments makes it difficult to acquire the related speciation in acidic and neutral conditions. The weak Sb(V)–O peak was only detected in strongly alkaline conditions. Fig. 2d shows the Fe 2p high-resolution spectra of the samples at pH 4, 7, and 11. The characteristic peaks of Fe(III)–O (∼714.00 eV, ∼727.16 eV) and Fe(II)–O (∼710.85 eV, ∼724.03 eV) were observed (Table S6). Moreover, two satellite peaks located at ∼718.86 eV and ∼732.49 eV were fingerprint regions of Fe(III) [51]. The high-resolution spectra of Sb 3d + O 1s and Fe 2p at pH 5.5, 8, and 10 are listed in Fig. S6 and Fig. S7. These results indicated that hydroxyl on the surface of iron oxyhydroxides played a significant role in enhancing the binding ability of Sb(V) through Fe–O–Sb coordination.

Extraction experiments were conducted to assess the Sb(V) content in the structure or on the surface. Fig. S8 illustrates that a higher amount of Sb(V) was adsorbed onto the Goe surface under acidic conditions. In contrast, upon the formation of Mag under strongly alkaline conditions, the surface Sb(V) content exhibited a significant decrease, comprising only 7.6% of the total. This observation suggested that a substantial portion of Sb(V) became incorporated into the iron mineral structure during the Fhy transformation. The structural incorporation of Sb(V) into iron-containing compounds notably reduced its release.

### 3.3 Spectral characteristics of Fhy transformation products

**Fig. 3:**
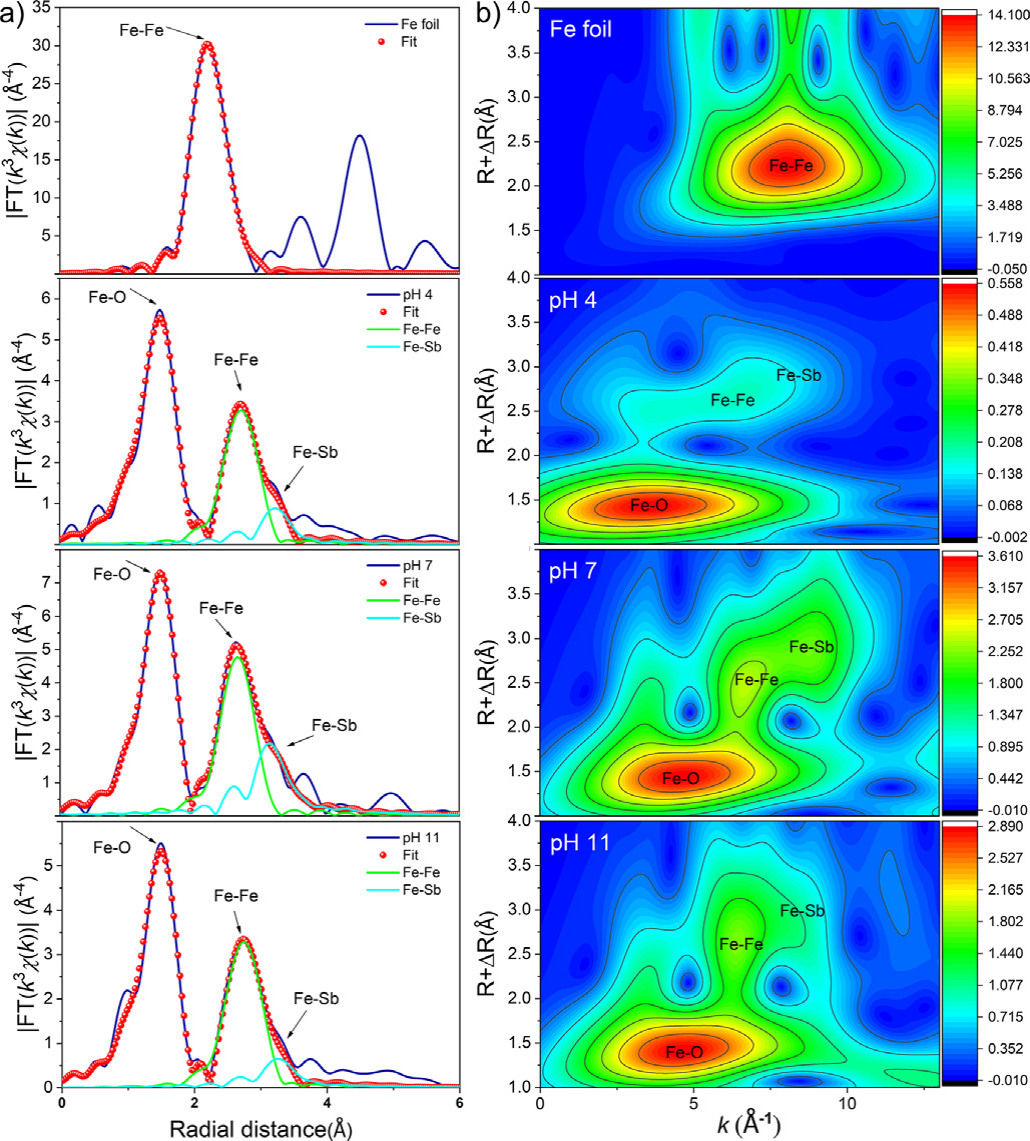
Morphological spectral characteristics of surface coordination between Sb(V) and transformed iron minerals. (a) Fe K-edge *k*^3^-weighted EXAFS spectra; (b) corresponding Fourier transform magnitude of solid-phase samples. EXAFS, extended X-Ray absorption fine structure.

The chemical coordination environment of iron atoms in the transformation products at pH 4, 7, 8, and 11 was characterized by Fourier transforms extended X-ray absorption fine structure (FT-EXAFS) and wavelet transform (Fig. 3). We used data of Fe for further analysis since the EXAFS data of Sb could not be obtained. Fe atom coordinating with Fe atom formed Fe–Fe bonds with a length of 2.47 Å. The Fe–Fe bonds could be observed in the conversion products at pH 4, 7, and 11, and these bonds were located in the second shell, while Fe–O and Fe–Sb coordination were located in the first and the third shell, respectively. The fitting distances of the Fe–O bond and Fe–Sb bond were 1.95–1.98 Å and 3.43–3.54 Å, respectively. The fitting results of Fe–O and Fe–Sb pathways showed that Sb(V) combined to the surface of the transformed products through the inner-sphere bidentate complexation, including edge-sharing and corner-sharing complexes in the process of IMPT [52]. The quantitative analysis of the first coordination (Fe–O) shell showed that all O atoms were at the same distance (1.95 Å−1.95 Å) from the central Fe at pH 4 and 11, which was consistent with the octahedral coordination of Fe and O [53]. By combining the results of TEM and XPS, it can be determined that the chemical coordination structure of iron contains Fe–O–Sb, conforming to inner-sphere bidentate complexation. The nearest neighbor structure around atoms, the information on atomic spacing, coordination number, and coordination element types are listed in Table S7.

FT-EXAFS fitting cannot be used to analyze the situation where several single or multiple scattering paths in coordination systems overlap in R space. Since the multiple scattering effect is a common overlapping phenomenon in FT-EXAFS, the transformed products are further analyzed to identify this effect by wavelet transform. The wavelet images of the transformed products were different from Fe foil, which meant that the coordination structure of the products was different from that of the Fe–Fe bond in Fe foil. Wavelet has a great resolution for the local element types of the central metal. Different colors represented the heights of the peak, and the products had metal element coordination with a mass fraction greater than Fe in the high K region, inferring the Sb(V) signal. According to EXAFS analysis, Fe–O, Fe–Fe, and Fe–Sb coordination existed in all transformation products, and the intensity of signal value followed an order of Fe–O > Fe–Fe > Fe–Sb (Fig. 3a). The weak signal of Fe–Sb coordination was mainly due to the low content of Sb(V) in the solid phase, which increased the detection difficulty. The Fe–Sb coordination tended to extend to the high-*k* region, which might be related to the scattering between Sb and Fe (Fig. 3b) [54].

### 3.4 Confirmation of the surface coordination of iron oxyhydroxides towards Sb(V)

**Fig. 4:**
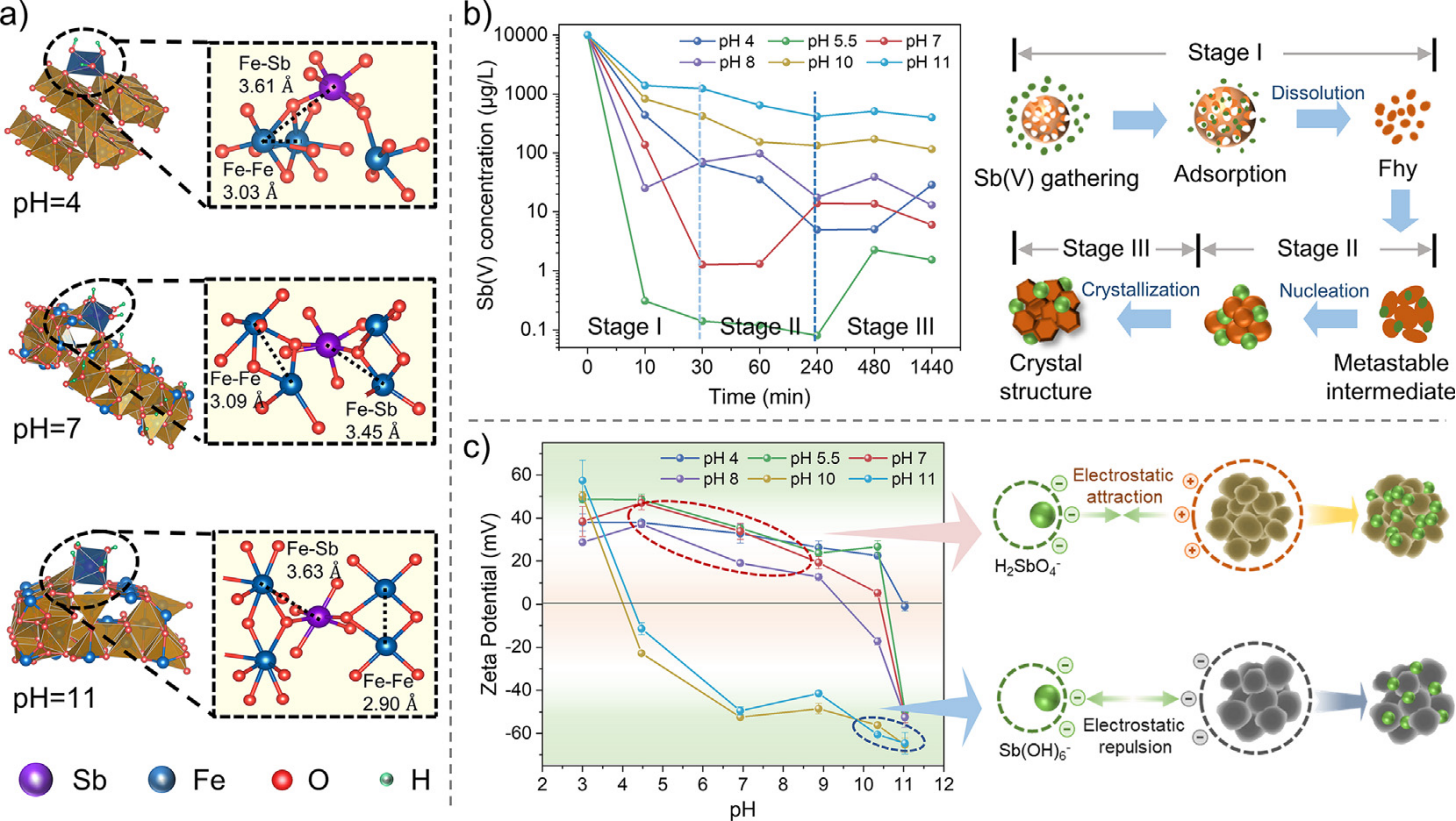
The removal mechanisms of Sb(V) in IMPT. (a) DFT-optimized inner-sphere bidentate surface configuration for Sb(V) combining with iron minerals; (b) changes in aqueous Sb(V) concentration over time; (c) the zeta potential of transformation products. IMPT, iron minerals phase transformation.

To elucidate the mechanism of interaction between Sb(V) and iron minerals, DFT and zeta potential were employed to confirm the cause of Sb(V) concentration attenuation. In this study, hydrogen bond interaction, inner-sphere bidentate binuclear complexation, and outer-sphere monodentate complexation were mainly considered. The optimized configuration was presented in Fig. 4a. Sb(V) combined with Goe, Hem, and Mag through the above three modes. The bond lengths of Fe–Sb in transformation products at pH 4, 7, and 11 were 3.61 Å, 3.45 Å, and 3.63 Å, respectively, consistent with the EXAFS results [46,55]. With increased pH, Fhy was converted into different secondary mineral, and the binding energy was significantly enhanced. The inner-sphere bidentate binuclear complexation had the largest contribution rate and the highest binding energy at different pH values (Fig. S9). Therefore, it was determined that Sb(V) combined with Goe, Hem, and Mag mainly through inner-sphere bidentate binuclear complexation, which was the most critical surface binding mechanism in this system [56]. More simulating calculation results were discussed in the supporting information (Fig. S10).

The changes of Sb(V) concentration attenuation over time, which could be considerably influenced by IMPT, were shown in Fig. 4b. It should be noted that due to the significant difference in the initial and final Sb(V) concentrations, the ordinate is expressed in logarithmic form. At the initial stage of the reaction, the significant decrease of Sb(V) concentration was attributed to the strong adsorption of Fhy itself and the formation of metastable intermediates with high activity during the phase transformation. It was speculated that prenucleation clusters and amorphous agglomerates were formed via the growth and aggregation of Fhy nanoparticles [57,58]. As the reaction time increased, the loss of surface-site density and highly active intermediates were induced by the dissolution of Fhy, which gradually nucleated, grew, and eventually recrystallized into more stable secondary minerals, causing the originally bound Sb(V) to be released by extrusion or desorption [59]. Under strong alkaline solutions, the concentration of Sb(V) tended to be stable, which was attributed to faster phase transformation and the formation of low-active Mag and Hem. Consequently, the adsorbed Sb(V) might be encapsulated in the crystalline phase before being released into the solution. In addition, the binding ability of Mag and Hem on Sb(V) was weaker than that of Fhy and other iron minerals, resulting in weak secondary adsorption of the transformation products. Therefore, the residual Sb(V) concentration in the aqueous solution was relatively high.

The dominant species of Sb and Fe were summarized and analyzed by Visual MINTEQ (Fig. S11). The zeta potentials of end products were determined (Fig. 4c). At pH 4–8, Sb(V) existed mainly in the form of H_2_SbO_4_^−^, and the positive charge on the iron oxyhydroxides surface rapidly combined with H_2_SbO_4_^−^ through strong electrostatic attraction, leading to a significant decrease in the concentration of Sb(V) [60]. At pH 10–11, the oxyhydroxide surface was negatively charged, while Sb(V) existed in Sb(OH)_6_^–^ species, indicating a certain electrostatic repulsion. This negative effect dramatically inhibited Sb–Fe binding, which might also be the dominant reason for the lower binding of Sb(V). Moreover, the formation of higher-density Mag at high pH led to the reduction of the contact area between iron solid and Sb(V), thus reducing the reaction rate and removal efficiency.

## 4 Environmental implications

**Fig. 5:**
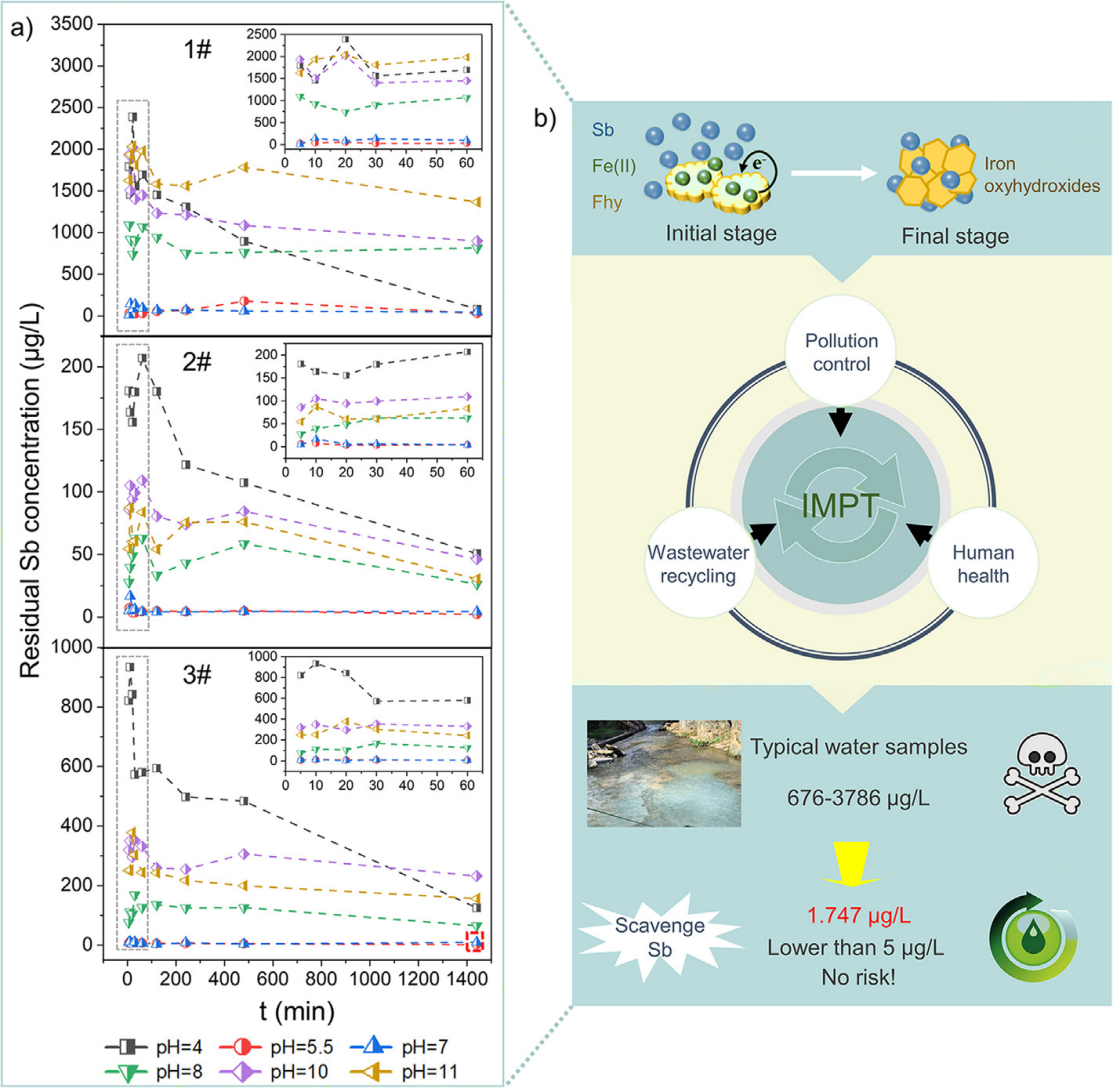
Beneficial impacts of IMPT process on Sb content. (a) residual Sb concentration with time during Fhy transformation in the natural water samples; (b) schematic diagram of the effect of the IMPT on Sb concentration in three representative water samples. 1#, 2#, and 3# represent the three typical water samples around Sb smelt sites, respectively.

To verify the critical role of IMPT in alleviating Sb pollution in natural water, three representative water samples around Sb smelter sites were examined (Fig. 5). Water quality parameters (concentration, turbidity, TOC, and pH) were measured, where the initial concentration of Sb reached thousands microgram per liter (676−3,786 μg/L) (Table S8). The residual Sb was barely detectable at pH 5.5 and 7 (only 1.747 μg/L), indicating that the recrystallization of Fhy to Goe was beneficial to the control of Sb contamination in natural water. However, the formation of Mag and Hem had no obvious merits in Sb removal (reach up to 1,366 μg/L) (Fig. 5a). These results indicate that the key pathway in IMPT could potentially reduce the Sb level to lower than 5 μg/L in these representative waters. Therefore, the IMPT process significantly contributed to wastewater pollution control due to the aforementioned mechanisms (electrostatic attraction, surface adsorption, and surface complexation). The IMPT process, with its high compatibility in the water environment, is beneficial to pollution control, wastewater recycling, and human health (Fig. 5b).

This study revealed that intensified electrostatic attraction and the formation of inner-sphere bidentate binuclear complexation contributed to the significant decrease of Sb(V), which provides new insights into PTEs pollution control and environmental risk mitigation. We also innovatively proposed the critical role of IMPT on Sb(V) removal in natural water, which helps to achieve ultra-low Sb(V) level. This finding indicated the profound and desirable impact on the control of PTEs in water environment. Specifically, this control occurred primarily during the early stages of IMPT and neutral conditions (pH 5.5–7), where a significant portion of the mobile Sb species incorporated into the structures of Fhy nanoparticles, resulting in the immobilization of PTEs within the mineral structure. However, it is important to note that under certain extreme environmental conditions, such as highly acidic conditions, the PTEs associated with the structural units of Fhy may potentially be released. In addition, in terms of PTEs level control, pivotal IMPT pathways and underlying mechanisms offer new perspectives to achieve safe water quality, which benefits mitigating the risk to human health and the ecological environment.

## Author contributions

X.Y.L.: data curation, visualization, writing–original draft. Y.Y.W., Z.L., L.Y.C.: conceptualization, methodology. H.R.X., J.H.W.: data curation, visualization. X.Y., W.C.Z.: conceptualization, methodology, writing–review & editing. All authors read and approved the final manuscript.

## Declaration of competing interests

The authors declare no competing financial interest.
