# Supplementary material for: Unveiling the crucial role of iron mineral phase transformation in antimony(V) elimination from natural water
Source: Eco Environ Health. 2023 Jul 24;2(3):176–83. doi: 10.1016/j.eehl.2023.07.006 (PMC10702924; doi:10.1016/j.eehl.2023.07.006)
Supplement: Multimedia component 1 [file mmc1.docx]

**Supplementary Material**

**Unveiling the crucial role of iron mineral phase transformation in antimony (V) elimination from natural water**

Xiaoyun Liu^a^, Yunyan Wang^a,b,c^, Hongrui Xiang^a^, Jiahui Wu^a^, Xu Yan^a,b,c,*^, Wenchao Zhang^a,b,c,*^, Zhang Lin^a,b,c^, Liyuan Chai^a,b,c^

^a^School of Metallurgy and Environment, Central South University, Changsha 410083, China

^b^State Key Laboratory of Advanced Metallurgy for Non-ferrous Metals, Changsha 410083, China

^c^Chinese National Engineering Research Center for Control & Treatment of Heavy Metal Pollution, Changsha 410083, China*Corresponding author

E-mail: [yanxu1202@csu.edu.cn](mailto:yanxu1202@csu.edu.cn); [wenchao.zhang@csu.edu.cn](mailto:wenchao.zhang@csu.edu.cn)

(24 pages, 11 Figures, 8 Tables)

**This file includes:**

Text S1. Characterization methods of the transformation products.

Text S2. Density functional theory (DFT) calculations.

Fig. S1. The XRD pattern of Fhy.

Fig. S2. SEM images of Fhy transformation products at different pH values.

Fig. S3. TEM images and EDS mapping of the transformation products at pH 7.

Fig. S4. TEM images and EDS mapping of the transformation products at pH 11.

Fig. S5. The survey XPS spectra of transformation products.

Fig. S6. The high-resolution spectra of Sb 3d+O 1s at pH 5.5, 8, and 10.

Fig. S7. The high-resolution spectra of Fe 2p at pH 5.5, 8, and 10.

Fig. S8. Sb(V) content extracted by Na_3_PO_4_ and HCl at different pH values.

Fig. S9. The proportion of hydrogen bond interaction, inner-sphere bidentate binuclear complex, and outer-sphere monodentate complex in different pH values.

Fig. S10. DFT optimized (a) hydrogen bond interaction, and (b) outer-sphere monodentate surface configuration for Sb(V) combining with iron minerals at pH 11.

Fig. S11. Species of Sb and Fe were simulated with Visual MINTEQ software.

Table S1. Basic parameters and phase analysis of Mossbaüer spectra of Fhy transformation products at different pH conditions.

Table S2. LCF result analysis of Fe K-edge XANES of Fhy transformation products at different pH conditions.

Table S3. Composition analysis of Fhy transformation products.

Table S4. EDS elemental content of transformation products at pH 7 and 11.

Table S5. The high-resolution spectra fitting parameters of Sb 3d+O 1s of transformation products.

Table S6. The high-resolution spectra fitting parameters of Fe 2p of transformation products.

Table S7. EXAFS fitting parameters at the Fe K-edge for Fhy transformation products.

Table S8. Water quality parameters of representative water samples.

**Text S1. Characterization methods of the transformation products.**

The mineral phase and crystal structure of prepared ferrihydrite (Fhy) and its transformation products were confirmed by A fully automated X-ray diffraction (XRD, Empyrean). A continuous scan mode was applied with Cu Kα radiation (40 kV and 40 mA). The scan parameters used were 10°-80° 2θ, with a step size of 0.0025° 2θ. The identification of peaks was analyzed with High-score software. Functional groups on the mineral surfaces were characterized by Fourier transform infrared (FTIR, Nicolet iS50) spectrometer. The samples were scanned in a range of 500-4000 cm^-1^ with a transmission mode. The data were processed using Omnic software. The relevant information on the valence states of elements and structural states of the samples was analyzed by X-ray photoelectron spectroscopy (XPS, ESCALAB 250Xi). The obtained XPS spectra were fitted using Avantage software. The morphologies of the samples were observed by using a scanning electron microscope (SEM, JSM-IT300LA) at an acceleration voltage of 20.0 kV with a gold spraying time of 2 min. The morphologies of the samples were characterized using high-resolution transmission electron microscopy (HRTEM, TF20), which can provide nanoscale elemental analysis. Particle morphology was observed in scanning TEM mode, and further elemental distribution was obtained using an energy dispersive spectrometer (EDS) with high-angle annular dark-field (HAADF) detectors for Fe, O, Sb, and K. The samples were measured at room temperature using a Germany Wissel iso-accelerated drive-type Mössbauer spectra with a radioactive source of ^57^Co(Pd). The spectra were fitted by the least square method.

The bonding of the products was analyzed using extended X-Ray absorption fine structure (EXAFS). First, the χ(k) exported from Athena was imported into the Hama Fortran code to calculate path-specific theoretical spectra to analyze the wavelet transform. The parameters were listed as follows: R range, 1-3.5 Å, k range, 0-13.0 Å^-1^, and the Morlet function with κ=10, σ=1 was used as the mother wavelet to provide the overall distribution. Fe foil, ferrihydrite, lepidocrocite, goethite, hematite, magnetite, maghemite, and potassium pyroantimonate (KSbH_6_O_6_) are used as standard samples. Fe foil is a standard material with the structural characteristics of iron and its compounds.

**Text S2. Density functional theory (DFT) calculations.**

The Vienna Ab Initio Package (VASP) was employed to perform all the density functional theory (DFT) calculations within the generalized gradient approximation (GGA) using the PBE formulation [1-3]. The projected augmented wave (PAW) potentials were chosen to describe the ionic cores and take valence electrons into account using a plane wave basis set with a kinetic energy cutoff of 450 eV [4]. Partial occupancies of the Kohn−Sham orbitals were allowed using the Gaussian smearing method and a width of 0.05 eV. The electronic energy was considered self-consistent when the energy change was smaller than 10^−4^ eV. A geometry optimization was considered convergent when the force change was smaller than 0.05 eV/Å.

**
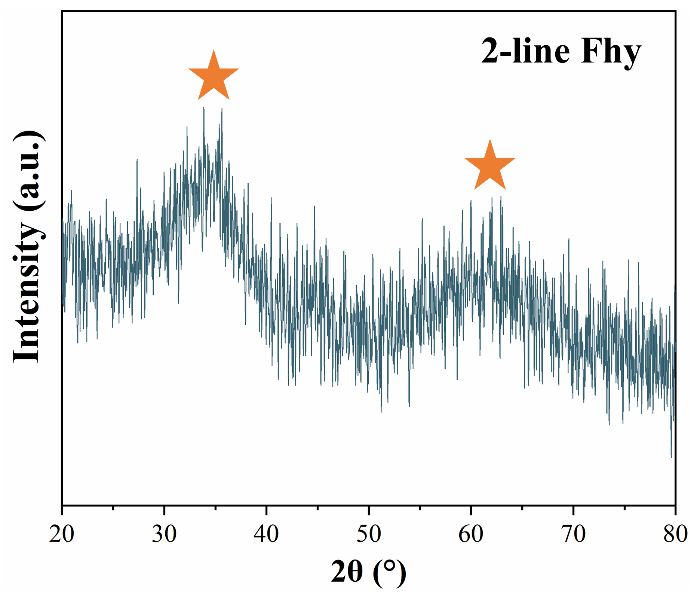
**

**Fig. S1.** The XRD pattern of Fhy.

**
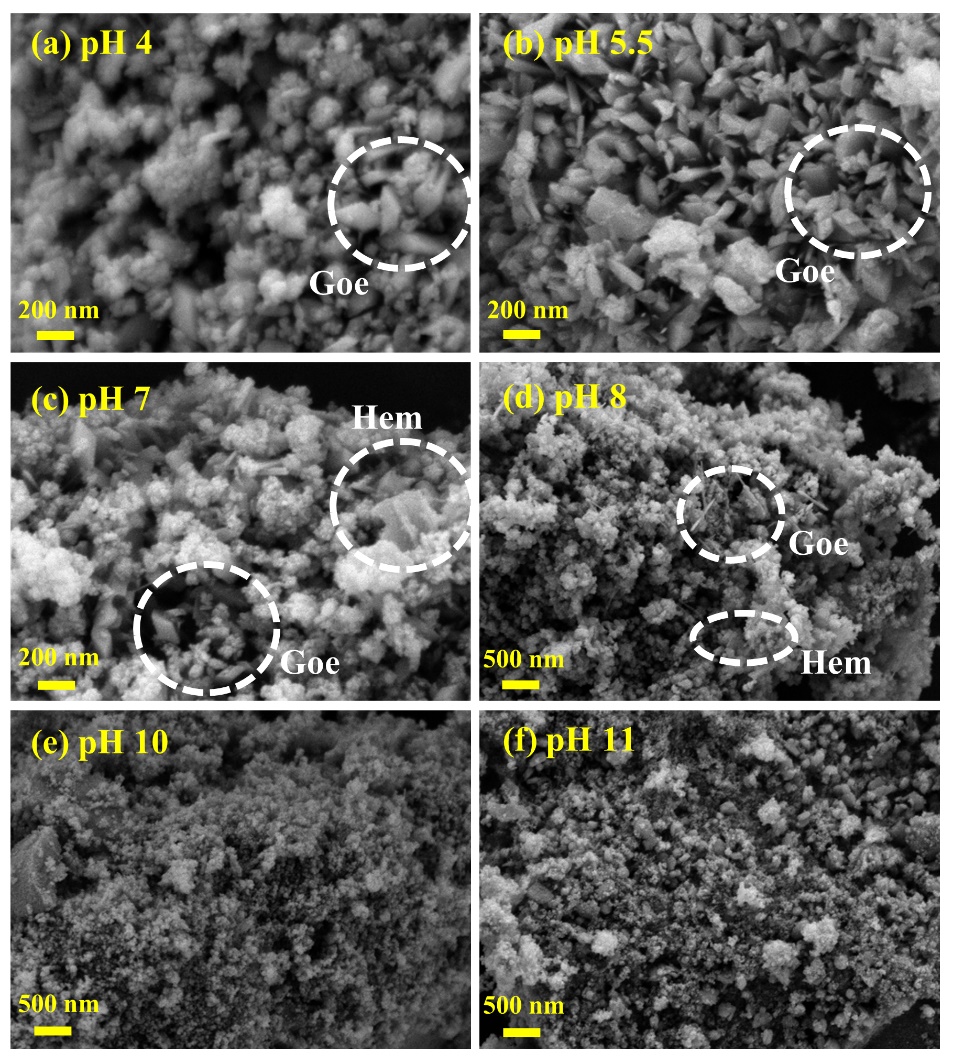
**

**Fig. S2.** SEM images of Fhy transformation products at different pH values. (a) pH 4, (b) 5.5, (c) 7, (d) 8, (e) 10, (f) pH 11. Goe and Hem represent goethite and hematite, representatively.

The morphologies of Fhy transformation products were observed using SEM. The obvious irregular needle shape of goethite particles can be observed at pH 4 and 5.5. Large amounts of rounded and platy particles were formed by magnetite and hematite accumulation at alkaline conditions, and their aggregation effect was well visible [5].

**
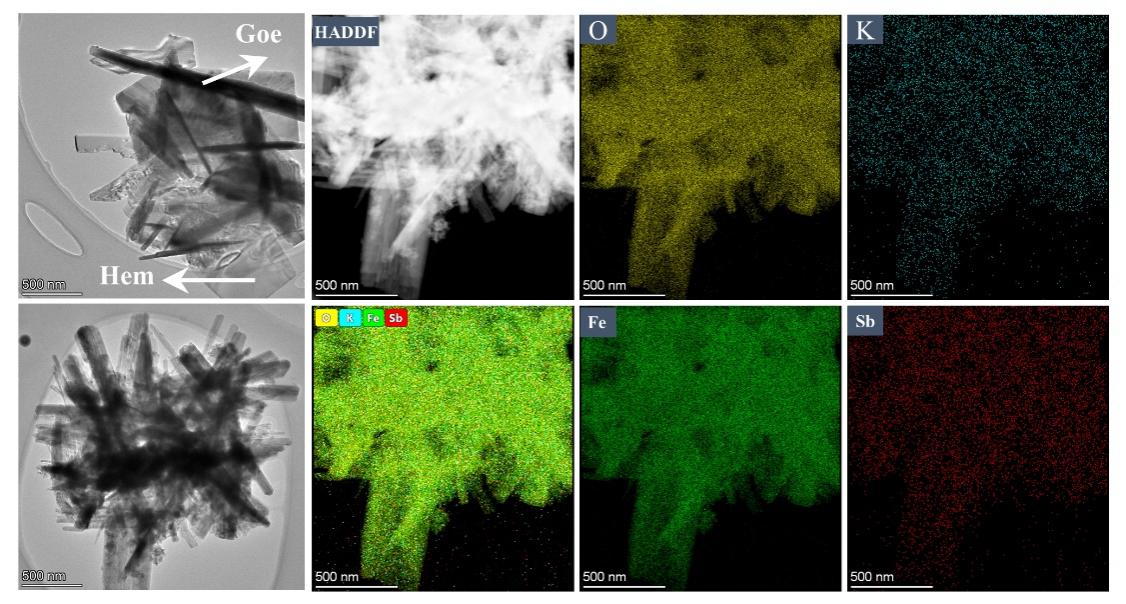
**

**Fig. S3** TEM images and EDS mapping of the transformation products at pH 7.

The acicular goethite was observed clearly. Hematite crystals were platy. The mapping results detected the elements of O, K, Fe, and Sb, among which O and Fe accounted for a large proportion, and the distribution of K and Sb was barely observed.

**
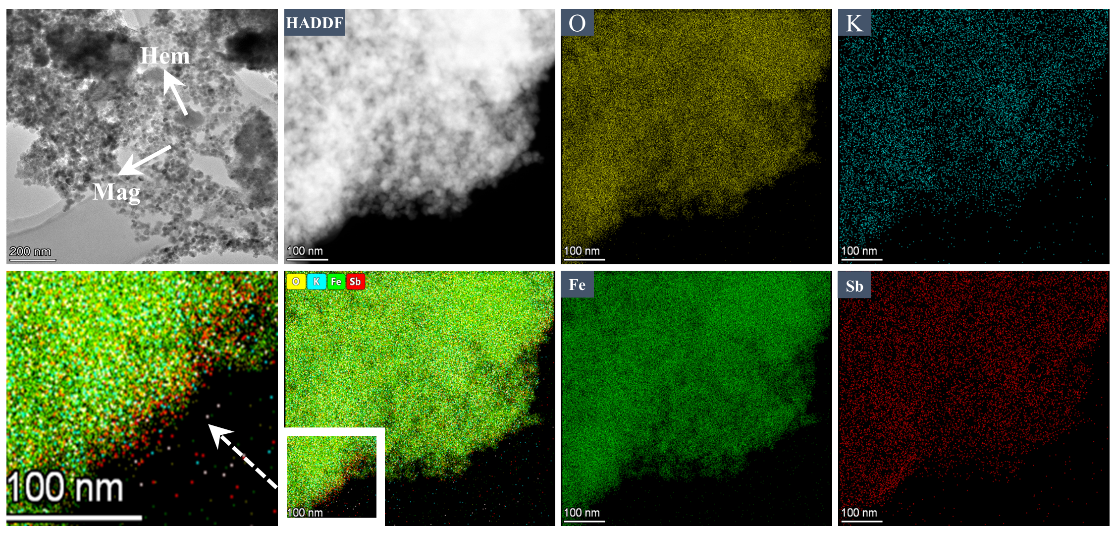
**

**Fig. S4.** TEM images and EDS mapping of the transformation products at pH 11.

Platy hematite crystals and rounded magnetite crystals were observed. The mapping detected the elements of O, K, Fe, and Sb. The distribution of Sb was observed in a small area tending to the edge.


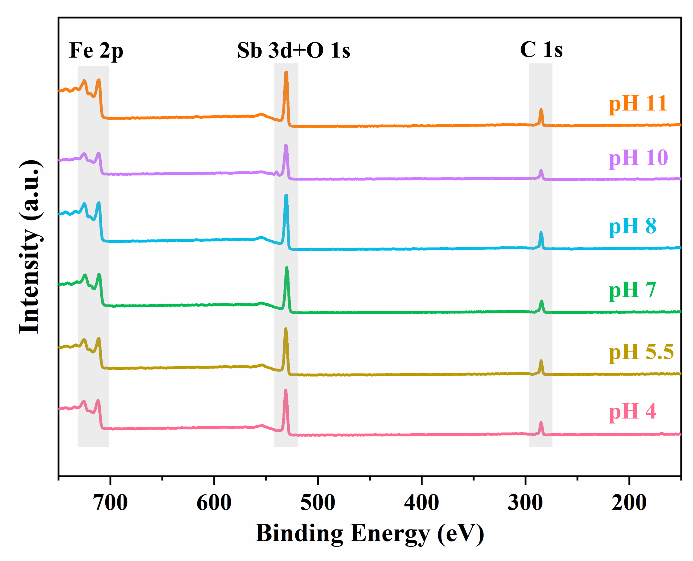


**Fig. S5.** The XPS survey spectra of transformation products.

The surface chemical composition and bonding characteristics of the transformation products were analyzed by XPS.


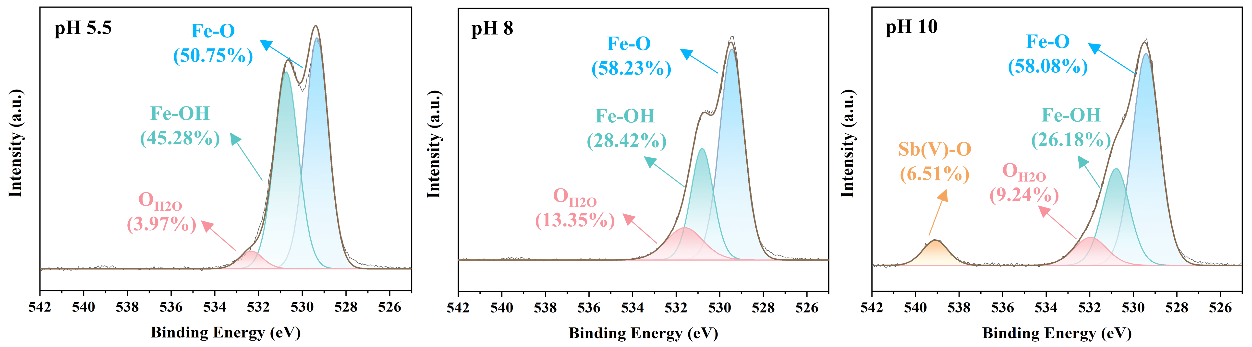


**Fig. S6.** The high-resolution spectra of Sb 3d+O 1s at pH 5.5, 8, and 10.

**
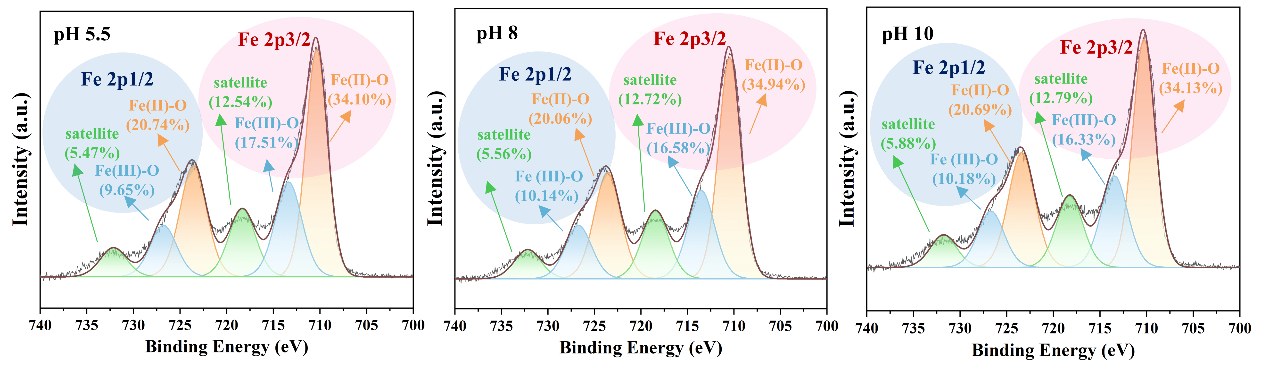
**

**Fig. S7.** The high-resolution spectra of Fe 2p at pH 5.5, 8, and 10.

As shown in Figure 2d, the Fe 2p high-resolution spectra where the binding energies at ~710 eV and ~723 eV were associated with Fe 2p_3/2_ and Fe 2p_1/2_. The results of Fe 2p spectra showed that Fe existed mainly in the state of +2 in the transformation products, and the difference in the composition and proportion of iron minerals.

**

**

**Fig. S8.** Sb(V) content extracted by Na_3_PO_4_ and HCl at different pH values.

The content of surface Sb(V) in the transformation products was lower than 20%. It is speculated that the content of more Sb(V) was incorporated into transformed iron oxyhydroxides.

**
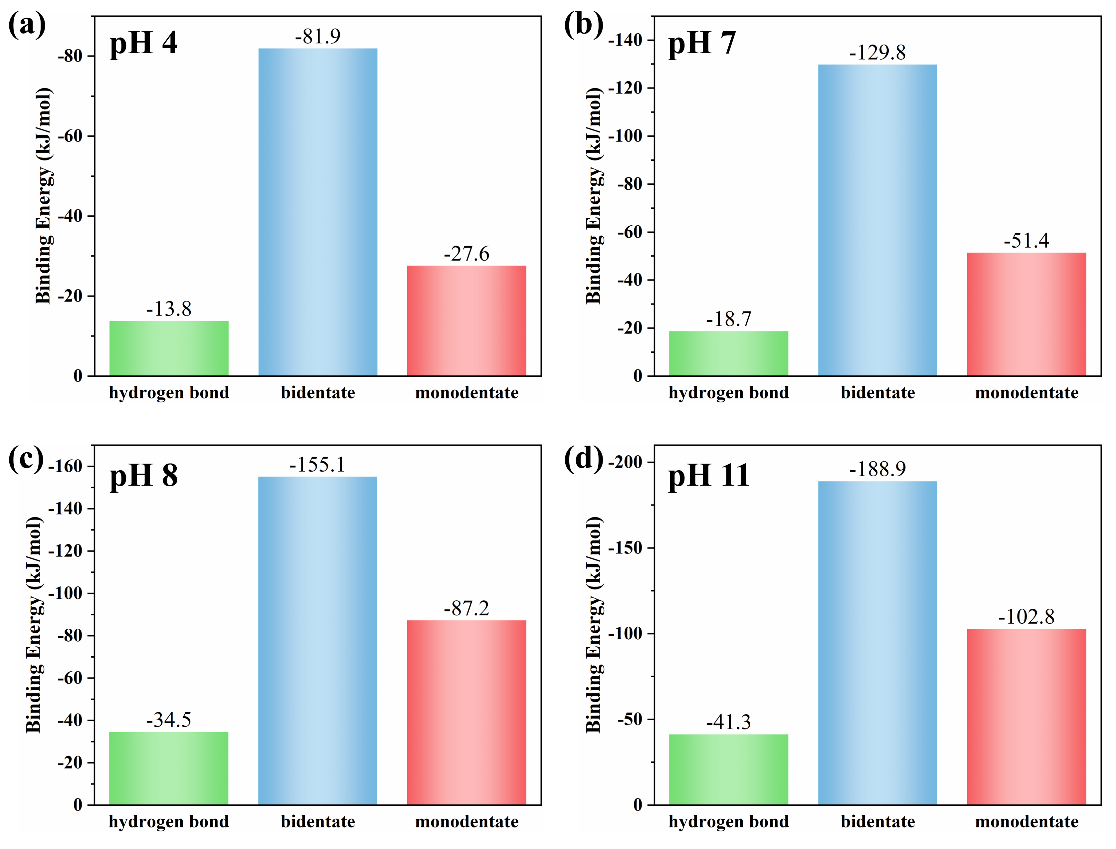
**

**Fig. S9.** The proportion of hydrogen bond interaction, inner-sphere bidentate binuclear complex, and outer-sphere monodentate complex in different pH values. (a) pH 4, (b) pH 7, (c) pH 8, and (d) pH 11.

At pH 4, 7, 8, and 11, the inner-sphere bidentate binuclear coordination has the largest contribution rate and the highest binding energy, which are −81.9 kJ/mol, −129.8 kJ/mol, −155.1 kJ/mol, and −188.9 kJ/mol, respectively.


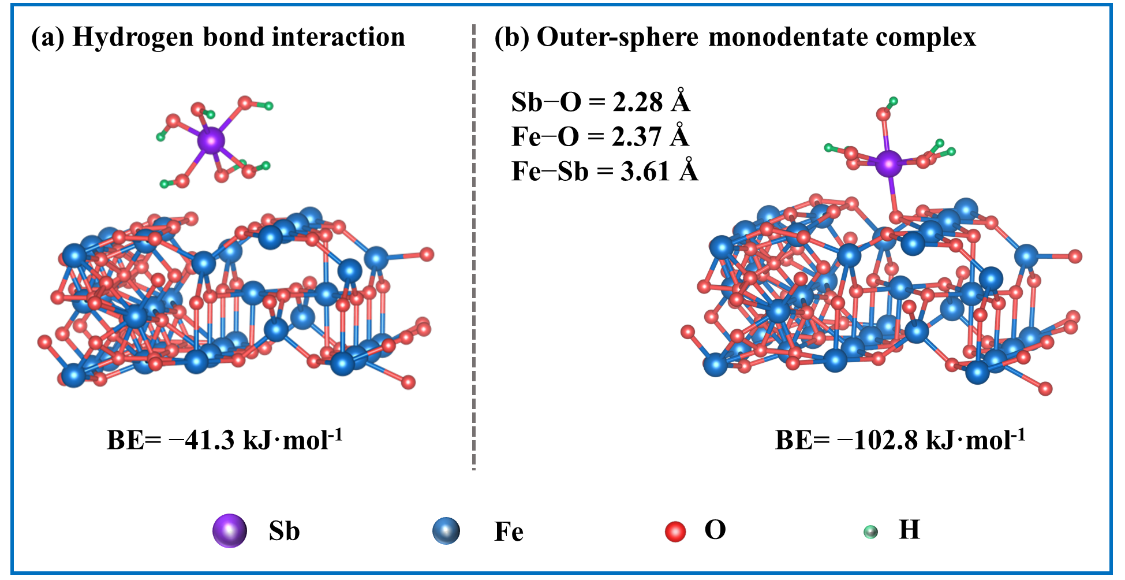


**Fig. S10.** DFT optimized (a) hydrogen bond interaction, and (b) outer-sphere monodentate surface configuration for Sb(V) combining with iron minerals at pH 11.

The most stable molecular configuration of Sb(OH)_6_^−^ combined with single-phase goethite and multiple phases (goethite and hematite, magnetite and hematite) was optimized by DFT simulation calculation.


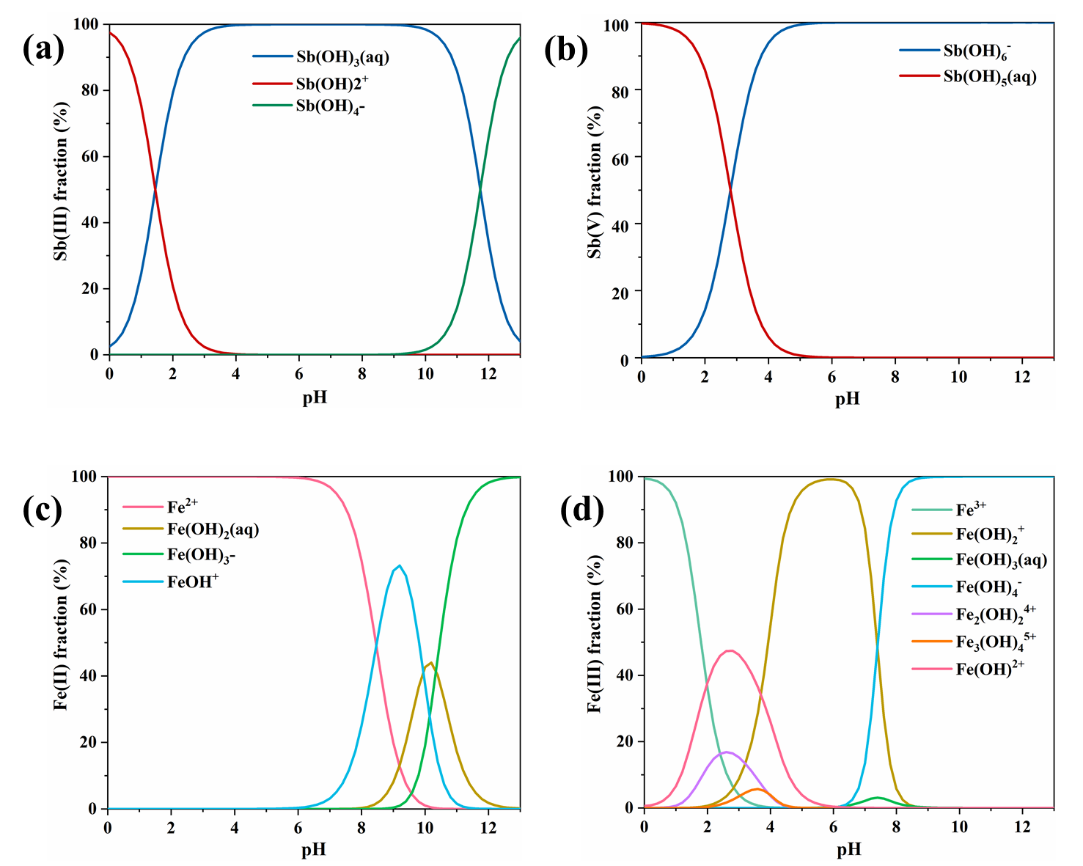


**Fig. S11.** Species of Sb and Fe in the (a) Sb(III)-H_2_O system, (b) Sb(V)-H_2_O system, (c) Fe(II)-H_2_O system, and (d) Fe(III)-H_2_O system were simulated with Visual MINTEQ software.

Sb(V) is mainly present as the negatively charged antimonate, and Fe(II) is a catalyst in the whole solution system.

**Table S1** Basic parameters and phase analysis of Mossbaüer spectra of Fhy transformation products at different pH conditions.

| Sample | Subspectrum | IS (mm/s) | QS (mm/s) | H (T) | Γ(mm/s) | Area(%) | Species |
| --- | --- | --- | --- | --- | --- | --- | --- |
| pH 4 | Sextet1 | 0.45 | -0.05 | 50.01 | 1.31 | 9.0 | Fe_2_O_3_ A-site |
|  | Sextet2 | 0.51 | 0.08 | 29.73 | 0.60 | 6.7 | *α*-FeOOH |
|  | Sextet3 | 0.38 | -0.05 | 37.7 | 0.70 | 14.6 | Fe_2_O_3_ B-site |
|  | Doublet | 0.34 | 0.67 | -- | 0.50 | 69.7 | *γ*-FeOOH |
| pH 7 | Sextet1 | 0.38 | -0.20 | 51.33 | 0.40 | 28.99 | Fe_2_O_3_ A-site |
|  | Sextet2 | 0.34 | -0.21 | 48.82 | 0.60 | 19.54 | Fe_2_O_3_ B-site |
|  | Sextet3 | 0.38 | -0.22 | 35.64 | 0.83 | 26.31 | *α*-FeOOH |
|  | Doublet | 0.36 | 0.56 | -- | 0.37 | 25.17 | *γ*-FeOOH |
| pH 8 | Sextet1 | 0.38 | -0.20 | 51.49 | 0.38 | 30.5 | Fe_2_O_3_ A-site |
|  | Sextet2 | 0.42 | -0.27 | 37.33 | 1.11 | 13.3 | *α*-FeOOH |
|  | Sextet3 | 0.35 | -0.21 | 49.00 | 0.62 | 20.9 | Fe_2_O_3_ B-site |
|  | Doublet | 0.34 | 0.66 | -- | 0.49 | 35.3 | *γ*-FeOOH |
| pH 11 | Sextet1 | 0.34 | 0.00 | 45.64 | 0.58 | 5.2 | Fe_2_O_3_ |
|  | Sextet2 | 0.34 | 0.01 | 48.79 | 0.58 | 4.8 | Fe_3_O_4_ |
|  | Doublet | 0.34 | 0.69 | -- | 0.51 | 90.1 | Fe(OH)_2_ |

IS, QS, H, and Γ denote isomer shifts, quadrupole splitting, hyperfine splitting field, and line width.

**Table S2** LCF result analysis of Fe K-edge XANES of Fhy transformation products at different pH conditions.

| Sample | Magnetite | Goethite | Hematite | Maghemite | Lepidocrocite | R-factor |
| --- | --- | --- | --- | --- | --- | --- |
| pH 4 | -- | 0.566 | 0.042 | -- | 0.391 | 0.0005 |
| pH 7 | 0.001 | 0.628 | 0.277 | 0.094 | 0.000 | 0.0019 |
| pH 8 | 0.000 | 0.604 | 0.243 | 0.153 | -- | 0.0019 |
| pH 11 | 0.274 | 0.040 | 0.394 | 0.293 | -- | 0.0001 |

**Table S3** Composition analysis of Fhy transformation products.

| Primary iron mineral | pH values | Transformation products |
| --- | --- | --- |
| ferrihydrite | Acidity (pH 4 and 5.5) | Main phase: goethite (α-FeOOH) |
|  |  | Trace phase: lepidocrocite (γ-FeOOH), hematite (α-Fe_2_O_3_), maghemite (γ-Fe_2_O_3_) |
|  | Neutral and weakly alkalinity (pH 7 and 8) | Main phase: goethite (α-FeOOH), hematite (α-Fe_2_O_3_) |
|  |  | Trace phase: lepidocrocite (γ-FeOOH), maghemite (γ-Fe_2_O_3_) |
|  | Strong alkalinity (pH 10 and 11) | Main phase: magnetite (Fe_3_O_4_), hematite (α-Fe_2_O_3_) |

**Table S4** EDS elemental content of transformation products at pH 7 and 11 (wt. %).

| Elements  products | O | K | Fe | Sb |
| --- | --- | --- | --- | --- |
| pH 7 | 36.74 | 0.24 | 62.64 | 0.38 |
| pH 11 | 32.32 | 1.50 | 65.15 | 1.03 |

**Table S5** The high-resolution spectra fitting parameters of Sb 3d+O 1s of transformation products.

| pH | 4 | | | 5.5 | | | 7 | | |
| --- | --- | --- | --- | --- | --- | --- | --- | --- | --- |
| parameters | Peak | FWHM | Areas | Peak | FWHM | Areas | Peak | FWHM | Areas |
|  | BE | eV | (%) | BE | eV | (%) | BE | eV | (%) |
| Sb 3d+O 1s: |  |  |  |  |  |  |  |  |  |
| O_H2O_ | 532.52 | 1.40 | 5.04 | 532.34 | 1.27 | 3.97 | 531.63 | 2.31 | 14.01 |
| Fe-OH | 530.78 | 1.31 | 41.78 | 530.73 | 1.31 | 45.28 | 530.75 | 1.39 | 37.00 |
| Fe-O | 529.48 | 1.40 | 53.18 | 529.33 | 1.25 | 50.75 | 529.41 | 1.27 | 48.99 |
| pH | 8 | | | 10 | | | 11 | | |
| parameters | Peak | FWHM | Areas | Peak | FWHM | Areas | Peak | FWHM | Areas |
|  | BE | eV | (%) | BE | eV | (%) | BE | eV | (%) |
| Sb 3d+O 1s: |  |  |  |  |  |  |  |  |  |
| Sb(V)-O | -- | -- | -- | 539.09 | 1.38 | 6.51 | 539.15 | 0.89 | 0.89 |
| O_H2O_ | 531.61 | 1.97 | 13.35 | 531.97 | 1.77 | 9.24 | 531.48 | 2.03 | 17.21 |
| Fe-OH | 530.81 | 1.22 | 28.42 | 530.79 | 1.46 | 26.18 | 530.71 | 1.22 | 13.86 |
| Fe-O | 529.45 | 1.32 | 58.23 | 529.42 | 1.48 | 58.08 | 529.35 | 1.41 | 67.94 |

**Table S6** The high-resolution spectra fitting parameters of Fe 2p of transformation products.

| pH | 4 | | | 5.5 | | | 7 | | |
| --- | --- | --- | --- | --- | --- | --- | --- | --- | --- |
| parameters | Peak | FWHM | Areas | Peak | FWHM | Areas | Peak | FWHM | Areas |
|  | BE | eV | (%) | BE | eV | (%) | BE | eV | (%) |
| Fe 2p: |  |  |  |  |  |  |  |  |  |
| satellite | 732.49 | 3.36 | 5.23 | 732.14 | 3.36 | 5.47 | 732.33 | 3.36 | 5.43 |
| Fe(III)-O | 727.16 | 3.36 | 9.76 | 726.78 | 3.36 | 9.65 | 726.75 | 3.36 | 9.64 |
| Fe(II)-O | 724.03 | 3.36 | 20.42 | 723.58 | 3.36 | 20.74 | 723.71 | 3.36 | 20.72 |
| satellite | 718.86 | 3.36 | 12.57 | 718.33 | 3.36 | 12.54 | 718.41 | 3.36 | 12.76 |
| Fe(III)-O | 714.00 | 3.36 | 15.90 | 713.36 | 3.36 | 17.51 | 713.25 | 3.36 | 17.83 |
| Fe(II)-O | 710.85 | 2.93 | 36.13 | 710.35 | 2.75 | 34.10 | 710.43 | 2.65 | 33.64 |
| pH | 8 | | | 10 | | | 11 | | |
| parameters | Peak | FWHM | Areas | Peak | FWHM | Areas | Peak | FWHM | Areas |
|  | BE | eV | (%) | BE | eV | (%) | BE | eV | (%) |
| Fe 2p: |  |  |  |  |  |  |  |  |  |
| satellite | 732.18 | 3.36 | 5.56 | 731.77 | 3.36 | 5.88 | 731.68 | 3.36 | 5.68 |
| Fe(III)-O | 726.71 | 3.36 | 10.14 | 726.70 | 3.36 | 10.18 | 726.58 | 3.36 | 9.71 |
| Fe(II)-O | 723.60 | 3.36 | 20.06 | 723.40 | 3.36 | 20.69 | 723.22 | 3.36 | 20.74 |
| satellite | 718.44 | 3.36 | 12.72 | 718.24 | 3.36 | 12.76 | 718.14 | 3.36 | 12.19 |
| Fe(III)-O | 713.52 | 3.36 | 16.58 | 713.45 | 3.36 | 16.33 | 713.37 | 3.36 | 15.59 |
| Fe(II)-O | 710.46 | 2.84 | 34.94 | 710.29 | 2.83 | 34.13 | 710.12 | 2.94 | 36.10 |

**Table S7** EXAFS fitting parameters at the Fe K-edge for Fhy transformation products.

| Sample | Shell | *CN^a^* | *R*(Å)*^b^* | *σ*^2^(Å^2^)*^c^* | Δ*E*_0_(eV)*^d^* | *R* factor |
| --- | --- | --- | --- | --- | --- | --- |
| Fe foil | Fe-Fe | 8* | 2.47 | 0.0047 | 7.7 | 0.0020 |
|  | Fe-Fe | 6* | 2.85 | 0.0068 |  |  |
| pH 4 | Fe-O | 6.4 | 1.97 | 0.0112 | -0.5 | 0.0079 |
|  | Fe-Fe | 4.5 | 3.07 | 0.0121 | -6.0 |  |
|  | Fe-Sb | 1.1 | 3.52 | 0.0079 | 9.3 |  |
| pH 7 | Fe-O | 7.4 | 1.99 | 0.0109 | 1.2 | 0.0047 |
|  | Fe-Fe | 7.0 | 3.03 | 0.0129 | 6.6 |  |
|  | Fe-Sb | 1.7 | 3.43 | 0.0049 | -6.6 |  |
| pH 8 | Fe-O | 7.1 | 1.98 | 0.0108 | 0.7 | 0.0134 |
|  | Fe-Fe | 7.3 | 3.00 | 0.0127 | 5.7 |  |
|  | Fe-Sb | 1.8 | 3.45 | 0.0045 | -4.1 |  |
| pH 11 | Fe-O | 5.6 | 1.95 | 0.0104 | -0.5 | 0.0125 |
|  | Fe-Fe | 5.3 | 3.08 | 0.0136 | -6.6 |  |
|  | Fe-Sb | 0.6 | 3.54 | 0.0059 | 10.5 |  |

*CN^a^*、*R*(Å)*^b^*, *σ*^2^(Å^2^)*^c^*, and Δ*E*_0_(eV)*^d^* denote the coordination number, inter-atomic distance, Debye-Waller factors, and energy shift, respectively.

**Table S8** Water quality parameters of representative water samples.

| Representative water samples | Sb(V) concentration  (μg/L) | pH | TOC  (mg/L) | Turbidity  (NTU) |
| --- | --- | --- | --- | --- |
| 1# | 3786 | 8.04 | 9.644 | 0.6 |
| 2# | 676 | 7.80 | 3.776 | 0.7 |
| 3# | 1689 | 7.73 | 3.865 | 1.4 |

**References**

[1] J.F. G Kresse, Efficient iterative schemes for Ab initio total-energy calculations using a plane-wave basis set, Physical Review B 54(16) (1996) 11169-11186.

[2] K.B. JP Perdew, M Ernzerhof, Generalized gradient approximation made simple, Phys. Rev. Lett. 77 (1996) 3865-3868.

[3] D.J. G. Kresse, From ultrasoft pseudopotentials to the projector augmented-wave method, Physical Review B 59(3) (1999) 1758-1775.

[4] P. Blöchl, Projector augmented-wave method, Physical Review B 50 (1994) 17953-17979.

[5] M. Tadic, D. Trpkov, L. Kopanja, S. Vojnovic, M. Panjan, Hydrothermal synthesis of hematite (α-Fe_2_O_3_) nanoparticle forms: Synthesis conditions, structure, particle shape analysis, cytotoxicity and magnetic properties, J. Alloys Compd. 792 (2019) 599-609.
